# Decomposition of changes in socioeconomic inequalities in catastrophic health expenditure in Kenya

**DOI:** 10.1371/journal.pone.0244428

**Published:** 2020-12-29

**Authors:** Purity Njagi, Jelena Arsenijevic, Wim Groot

**Affiliations:** 1 United Nations University-MERIT, Maastricht Graduate School of Governance, Maastricht University, Maastricht, Netherlands; 2 Faculty of Law, Economics and Governance, School of Governance, Utrecht University, Utrecht, Netherlands; 3 Faculty of Health, Medicine and Life Sciences, Department of Health Services Research, Maastricht University, Maastricht, Netherlands; Tulane University, UNITED STATES

## Abstract

**Background:**

Catastrophic health expenditure (CHE) is frequently used as an indicator of financial protection. CHE exists when health expenditure exceeds a certain threshold of household consumption. Although CHE is reported to have declined in Kenya, it is still unacceptably high and disproportionately affects the poor. This study examines the socioeconomic factors that contribute to inequalities in CHE as well as the change in these inequalities over time in Kenya.

**Methods:**

We used data from the Kenya household health expenditure and utilisation (KHHEUS) surveys in 2007 and 2013. The concertation index was used to measure the socioeconomic inequalities in CHE. Using the Wagstaff (2003) approach, we decomposed the concentration index of CHE to assess the relative contribution of its determinants. We applied Oaxaca-type decomposition to assess the change in CHE inequalities over time and the factors that explain it.

**Results:**

The findings show that while there was a decline in the incidence of CHE, inequalities in CHE increased from -0.271 to -0.376 and was disproportionately concentrated amongst the less well-off. Higher wealth quintiles and employed household heads positively contributed to the inequalities in CHE, suggesting that they disadvantaged the poor. The rise in CHE inequalities overtime was explained mainly by the changes in the elasticities of the household wealth status.

**Conclusion:**

Inequalities in CHE are persistent in Kenya and are largely driven by the socioeconomic status of the households. This implies that the existing financial risk protection mechanisms have not been sufficient in cushioning the most vulnerable from the financial burden of healthcare payments. Understanding the factors that sustain inequalities in CHE is, therefore, paramount in shaping pro-poor interventions that not only protect the poor from financial hardship but also reduce overall socioeconomic inequalities. This underscores the fundamental need for a multi-sectoral approach to broadly address existing socioeconomic inequalities.

## Introduction

Healthcare systems’ reliance on out-of-pocket (OOP) payments can impose a financial burden on households, preventing some from seeking care and turning catastrophic for those who do [1]. It is estimated that by 2010, 808 million people in the world experienced catastrophic costs, and another 97 million ($1.90-a-day poverty line) were impoverished due to health care payments [2]. This is more profound in developing countries where OOP payments are a dominant feature of financing health care [3]. Catastrophic health expenditure (CHE) has been widely used as an indicator of the extent to which the health system protects households from healthcare-related financial hardship [4]. CHE refers to any expenditure on health that threatens a household’s financial ability to maintain its subsistence needs, and this does not necessarily refer to high costs [5]. Therefore, catastrophic health expenditure occurs when OOP payments exceed a certain threshold of household consumption [6]. The poorest households are most at risk, given that even a small amount of health spending can have catastrophic effects [7]. Other groups vulnerable to CHE include households with chronically ill members, older members, and/or children [8,9].

In Kenya, health financing includes OOP payments for several services at different levels of care [10]. These payments have been shown to be regressive [11]. CHE is still prevalent in Kenya [12] in spite, of the abolition of user fees at some levels of healthcare, such as maternity care and services for children under five. Moreover, 7.1% of Kenyans are reported to have incurred CHE in 2018 [13]. Although Kenya has a National Hospital Insurance Fund (NHIF), its coverage and package are limited. The NHIF has not been able to reach out to the majority of Kenyans households and individuals, especially the poor and those in the informal sector [14–16]. Furthermore, up until 2014, the NHIF package only covered inpatient services, and even the new insurance scheme is based on premiums under a contributory and voluntary mechanism; hence, the poor may not be able to pay [17].

Kenya is characterised by a high level of inequalities in comparison to its neighbouring East African counterparts, Uganda and Tanzania. These inequalities manifest in different forms and sectors, including health, and are observed across regions, genders, and even specific segments of the population [18,19]. The health care system has been criticised for regional discrepancies in health service distribution, disparities in resource allocations, and inequitable access to quality health services [20]. Furthermore, inequalities in access to healthcare services also exist, with the poorest forgoing essential services due to financial burden [21].

Several studies have assessed the magnitude of CHE in Kenya and, to a limited extent, the inequalities in CHE [22,23]. Nevertheless, these studies have not assessed the determinants of these inequalities and the change in inequalities over time. Evidence suggests that inequalities in CHE exist and are disproportionately concentrated amongst particular groups [24,25]. In essence, catastrophic payments are more severe than they appear when presented as plain fractions of the population, as this can mask the fact that the poor are more likely to exceed this threshold [26].

This study aims to assess the factors that influence inequalities in CHE and the change in these inequalities over time, i.e., from (2007 to 2013). Applying decomposition approaches, we add to the existing literature on CHE in Kenya and the region in two ways. First, we provide empirical evidence on the underlying determinants of inequalities in CHE. Second, we assess the change in CHE inequalities over time and the associated factors. Furthermore, in addition to the magnitude of inequalities, policymakers and researchers’ are also interested in understanding the potential causes of socioeconomic inequalities [27]. A trend analysis of the changes in inequalities over time may also reveal whether current inequalities are newly emerging or persistent problems [28]. Hence, the findings may inform the formulation of policies and actions aimed at reducing the existing inequalities in Kenya and its regions.

## Materials and methods

### Data source and sample

This study utilises data from two rounds (2007 and 2013) of the Kenya household health expenditure and utilisation surveys (KHHES). These are nationally representative cross-sectional surveys implemented by the Ministry of Health (MOH) in collaboration with the Kenya National Bureau of Statistics (KNBS). The surveys collect data on various aspects of health, including the following factors.

#### Health and household expenditure data

This includes outpatient costs and routine health expenses in the 4 weeks preceding the survey, inpatient costs in the last one year, food expenditure in the last one month (2007) and last seven days (2013), monthly household expenditure in the last one month, and annual household expenditure.

#### Utilisation of outpatient and inpatient services

This includes access to health insurance, individual reasons for not seeking care when ill, the type of provider where care was sought, the mode of payment for services received and funds sources.

#### Demographic and socioeconomic characteristics

This includes the wealth status of the household, number of household members, education level of household members, age of household members, the gender of household members, and employment status of the household head.

The two samples are drawn from the national master sampling frame, the National Sample Survey and Evaluation Program (NASSEP). A new sample is drawn in each year using a multi-stage stratified sampling design. The 2007 wave surveyed a total of 8,453 households from 737 clusters, of which 506 (68.7%) were rural, and 231(31.3%) were urban clusters. The 2013 wave surveyed a total of 33,675 households drawn from 1,347 selected clusters, of which 814 (60%) were rural and 533 (40%) were urban clusters. The difference in the sample sizes between the two rounds of the survey is because, while the 2007 sample was representative at the national level, 2013 was representative at both the national and sub-national (county) level to cater to the newly created sub-national structure of governance in Kenya as per the new constitution of 2010. Both samples provide population weights at the household level.

The sample data utilised in the analysis of this study is limited to those who sought care when ill, and the CHE is based on the OOP payments for health services received. This includes 3,728 households drawn from 737 clusters in 2007 and 16,526 households drawn from 1,347 clusters in 2013. We use the adjusted survey weights to account for the different survey samples.

### Variables

The main variable of interest (dependent) in this study is the incidence of CHE, which is a measure of the OOP payment relative to the household capacity to pay [29]. The commonly documented drivers of CHE are used as the independent variables [9,30,31]. These drivers include the following:

1. Demographic characteristics of the household (gender of the household head, age group of the household head, households with children under five years, households with elderly members, household size).
2. Socioeconomic characteristics of the households (wealth status, education level of the household head, employment status of the household head).
3. Geographical characteristic of the household (urban/rural residence)
4. Healthcare access factors (households with a chronically ill member, health insurance status, type of health provider).

### Data analysis

#### Measuring the incidence of catastrophic health expenditure

This study calculated CHE using the approach proposed by XU [32]. Xu estimates the incidence of catastrophic payments from the reported OOP payments as a share of 40% total non-food expenditure, also known as capacity to pay (CTP) [29]. This approach has been proposed by WHO and defines CTP as income remaining after subsistence needs have been met [8]. To estimate CHE using this approach, we use data on OOP payments and household consumption expenditure, including food and non-food expenditure. OOP payments include both medical costs for inpatients and outpatients, such as consultation, diagnosis, drugs and admission costs, and non-medical costs for inpatient and outpatients such as transport. OOP payments are net of costs not paid directly by the patients, including those paid through insurance, exemptions, and reimbursements.

Household (consumption) expenditure was calculated using the expenditure on food and non-food items. Food expenditure refers to how much the household spent on food and beverages items, excluding alcoholic beverages and food consumed outside the house. Non-food expenditure refers to recurring monthly expenditure such as rent and utilities, and annual expenditure such as house maintenance and education/fees, and capital expenditure, such as the purchase of assets.

To standardise and convert the costs into a common reference period of a year (annual), we annualised the outpatient costs by multiplying the total costs by 13, given the costs relate to the last four weeks preceding the survey. This is also consistent with other studies in Kenya [12,13,23].

We generated equivalised food expenditure by dividing each household’s food expenditure by the equivalised household size. Equivalised household size is used to reflect household composition and size based on a scale of 0.56 [29,33]. We identified the food expenditure shares of total household expenditure that are at the 45th (food45) and 55th (food55) percentiles across the whole sample. We then obtained the weighted average of food expenditure in the 45th to 55th percentile range. This gave the subsistence expenditure per (equivalent) capita, which is also the poverty line (pl).

$$\text{pl}=\;\frac{\sum {\text{w}}_{\text{h}}{*\text{eqfood}}_{\text{h}}}{\sum {\text{w}}_{\text{h}}}$$ (1)

Where, w_h_ is the equivalised household size in the 45^th^ and 55^th^ percentile. The subsistence expenditure for each household (se_h_) was derived by the following formula:

$${\text{se}}_{\text{h}}={\text{pl}\;*\;\text{eqsize}}_{\text{h}\;}$$ (2)

Household CTP, which is the household non-subsistence spending, was calculated using the following formula:

$$\text{CTP}=\{\begin{array}{l} exp_{h}-{\text{se}}_{\text{h}}\;\;\;\;\text{if}\;\;\;\;{\text{se}}_{\text{h}}\leq food_{h}\\ exp_{h}-food_{h}\;\;\;\text{if}\;\;\;{\text{se}}_{\text{h}}>food_{h} \end{array}$$ (3)

Where, *exp*_*h*_ is the household expenditure, and *food*_*h*_ is the food expenditure.

CHE binary variable was then created based on the fraction of OOP divided by CTP (OOPCTP), in which CHE took the value 1 if the fraction was greater or equal to 0.4 and 0 if otherwise.

#### Measuring inequality in catastrophic health expenditure

We used the concentration index (CI) to measure the extent of socioeconomic-related inequalities in CHE for 2007 and 2013. The CI has been extensively applied to quantify the extent of socioeconomic-related inequalities in health variables [34]. The CI measures the extent to which the health variable differs across individuals ranked by the socioeconomic indicator [35]. There are debates on the most suitable concentration index approach, but there is no consensus on which concentration index is ‘superior’ to the others [36,37]. However, there is an emphasis that a suitable index should satisfy specific fundamental properties including, i) cardinal invariance: A linear transformation of the health variable does not affect the index value, ii) transfer: A small transfer of the health variable from a richer to a poorer individual translates into a pro-poor change in the concentration index, and iii) mirror: the CIs of the presence of the health variable and absence of the health variable should be mirror images of each other [38].

The concentration index depends on the relationship between the health variable and the rank of the socioeconomic variable [26]. We classified the households into socioeconomic quintiles using per capita consumption expenditure to create a variable that ranks the households by their consumption (expenditure) status from the poorest to the richest quintiles. The concentration index was derived as follows:

$$\begin{array}{c} \text{CI}=\;\frac{2}{\mu_{\text{h}}}\sum_{\text{i}=1}^{\text{n}}({\text{h}}_{\text{i}}-\mu_{\text{h}})(R_{\text{i}}-\;\frac{1}{2})\\ =\frac{2}{\mu_{\text{h}}}\text{cov}(\text{h},\;R) \end{array}$$ (4)

where n denotes the number of observations, h_i_ is the health variable(CHE), μ is the mean of h, and ${\text{R}}_{i}-\frac{1}{2}$ is the fractional socioeconomic rank ranging from the poorest to the richest.

The concentration index ranges between −1 to +1, with zero(0) value meaning no socioeconomic-related inequality. The concentration index is intended to show the direction of the relationship between CHE and the socioeconomic variable, and the degree of variability in the distribution of CHE [26].

### Decomposition methods

#### Decomposition of the concentration index

To assess the relative contributions of each factor to inequalities in CHE, we decomposed the concentration index of CHE into its contributory factors using the Wagstaff approach [39]. This is the dominant decomposition procedure that has been comprehensively applied to explore the determinants of the socioeconomic gradient [27,40].

First, the concentration index of CHE was calculated, then that of each of the contributory factors. Second, we calculated the absolute contribution of each factor (χ) to the concentration index of CHE (y). A linear regression model is assumed to link the health variable CHE (y) to a set of factors (c_k_) as follows:

$$\text{y}=\alpha \;+\sum_{k}\beta_{k}X_{k}+\varepsilon$$ (5)

Given the relationship between y and χ in Eq (5), the concentration index for y, can be written as:

$$\text{C}=\;\sum_{\kappa }(\frac{\beta_{\kappa }{\bar{X}}_{\kappa }}{\mu }\;){\text{C}}_{\kappa }+\frac{GC_{\varepsilon }}{\mu }$$ (6)

where μ is the mean of y, ${\bar{X}}_{\kappa }$ is the mean of χ _k_ (k set of factors), c_k_ is the concentration index for χ _k_ (defined analogously to C), and GC_ε_ is the generalised concentration index for the error term (ε). $\left(\frac{{Β}_{\kappa }{\bar{X}}_{\kappa }}{\mu }\;\right)$ is the elasticity indicating the impact of each factor on the outcome(y) [26].

The results are intended to show the CI for each of the explanatory variable, the elasticities, the absolute and percentage contribution of each variable. A negative concentration index indicates that the variable had pro-poor distribution, while a positive concentration index indicates the variable had a pro-rich distribution. The absolute contribution refers to the contribution of the explanatory variables to the overall CHE inequalities. The absolute contribution is the product of the elasticity and the partial concentration index of each of the explanatory variables. A positive absolute contribution shows that the variable favoured the worse-off, whereas a negative absolute contribution shows that the variable favoured the better-off.

#### Oaxaca-type decomposition of change in concentration index

We applied the Oaxaca-type decomposition [26] to estimate the change in CHE inequalities between 2007 and 2013.

$$\Delta \text{C}=\;\sum_{\kappa }\eta_{\kappa t}({\text{C}}_{\kappa t}-{\text{C}}_{\kappa t-1})+\sum_{\kappa }{\text{C}}_{\kappa t-1}(\eta_{\kappa t}-\eta_{\kappa t-1})+\Delta (\frac{{\text{GC}}_{\varepsilon t}}{\mu_{t}})$$ (7)

where t refers to time period and Δ denotes first differences.

In Eq (7), we weighted the difference in concentration indices (C_κt_ − C_κt−1_) by the second-period elasticity η_κt_ and weighted the difference in elasticities (η_κt_ − η_κt−1_) by the first-period concentration index C_κt−1_.

An alternative to Eq (7) would be to weight the difference in concentration indices (C_κt_ − C_κt−1_) by the first-period elasticity η_κt−1_ and weight the difference in elasticities (η_κt_ − η_κt−1_) by the second-period concentration index C_κt_ as expressed in Eq (8) [26].

$$\Delta \text{C}=\;\sum_{\kappa }\eta_{\kappa t-1}({\text{C}}_{\kappa t}-{\text{C}}_{\kappa t-1})+\sum_{\kappa }{\text{C}}_{\kappa t}(\eta_{\kappa t}-\eta_{\kappa t-1})+\Delta (\frac{{\text{GC}}_{\varepsilon t}}{\mu_{t}})$$ (8)

This explains the amount of change in CHE inequalities that was due to variations in changes in the unequal distribution of determinants (ΔC) or the elasticities of determinants (Δη). ΔC.η_kt_ and ΔC.η_kt-1_ shows the changes in the amount of inequalities in the determinants, whereas Δη.C_kt-1_ and Δη.C_kt_ shows the changes in elasticities of the determinants. A positive sign means that the variable contributed to an increase in inequality, whereas a negative sign shows that the variable reduced the inequality over time.

All analyses were conducted in STATA 14/SE.

## Results

### Descriptive analysis of households experiencing catastrophic health expenditure

**Fig 1:**
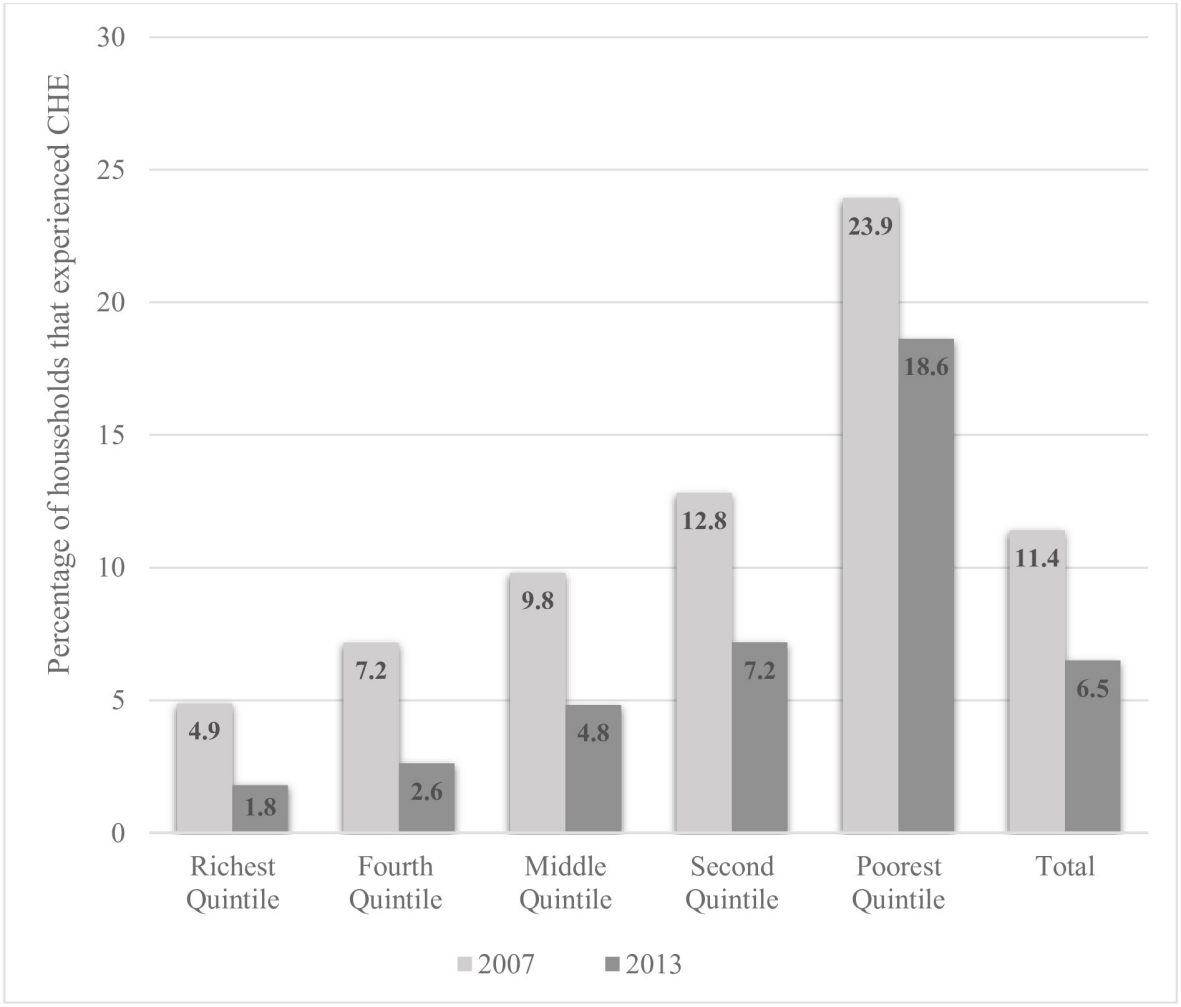
Incidence of catastrophic health expenditure by wealth quintiles, 2007 and 2013.

First, we computed the incidence of CHE, which showed a decrease from 11.4% in 2007 to 6.5% in 2013 at a threshold of 40% CTP. Similarly, we noted a decline in CHE across all the wealth status quintiles between 2007 and 2013. In absolute terms, the decline was greater among the lower wealth status groups; however, as percentage change, the richest group experienced a higher decline in CHE relative to other wealth categories. These analyses are presented in the S1 Table. Fig 1 presents the overall incidence of CHE and the distribution of CHE across the five wealth quintiles in 2007 and 2013.

The distribution of households facing CHE by the various socioeconomic characteristics in 2007 and 2013 is presented in Table 1. The analysis shows an increase in the percentage of households facing CHE among the lower wealth quintiles and a decline in households facing CHE among the higher wealth quintiles. For instance, the poorest quintile increased from 26.5% to 34.3%, while the richest quintile decreased from 15.7% to 9.2% in 2007 and 2013 respectively.

**Table 1:** Socioeconomic characteristics of the households that experienced catastrophic health expenditure.

Variables	Categories	2007			2013	
		N (427)	Percent (%)	N (1084)		Percent (%)
Wealth status	Poorest quintile	162	26.5	547		34.3
	Second quintile	99	18.5	225		21.7
	Middle quintile	74	16.9	152		18.7
	Fourth quintile	55	22.5	92		16.2
	Richest quintile	37	15.7	68		9.2
Gender of household head*	Female head	288	70.9	729		67.2
	Male head	118	29.1	355		32.7
Age group of households’ head*	Below 25 years	20	4.9	41		3.8
	25–40 years	156	38.3	372		34.3
	40+ years	231	56.8	671		61.9
Education of household head*	No Education	150	36.9	11		1.0
	Primary education	159	39.1	805		74.4
	Secondary education	82	20.2	178		16.4
	Tertiary education	16	3.9	88		8.1
Employment status of household head*	Unemployed HH head	169	41.5	258		23.8
	Employed HH head	238	58.5	826		76.2
Household Size	1–3 Small household	219	51.3	356		32.8
	4–6 Medium household	134	31.4	502		46.3
	7+ Large household	74	17.3	226		20.8
Household with U5 Children	Above 5 years	287	67.2	573		52.9
	Below 5 years	140	32.8	511		47.1
Elderly (Above 60+)	Below 60 years	294	68.9	687		63.4
	Above 60 years	133	31.2	397		36.6
Health insurance	Insured household	353	82.7	803		74.2
	Not insured	74	17.3	281		25.9
Residence	Rural residence	331	77.5	752		69.4
	Urban residence	96	22.5	332		30.6
HH with member wt. chronic illness	No chronic illness	291	68.3	550		50.7
	Chronic illness	135	31.7	534		49.1
Type of health provider*	Public provider	192	45.4	437		40.4
	Private provider	151	35.6	491		45.4
	Other providers	80	18.9	154		14.2

*Dropped missing cases (<20) in 2007.

The majority of the households that experienced CHE were female-headed, had a less educated (primary level and below) household head, had an employed household head, and the household head was above 40 years. We also observed that the majority of the households that experienced CHE had children under five years, an elderly member, were not insured, lived in rural areas, and had a chronically ill member. However, we also observed that in the 2007 sample, the majority of households that experienced CHE were from small-sized households, whereas in 2013, the majority were from medium-sized households. The results further suggest that the majority of households that experienced CHE in 2007 had sought care from a public health provider, whereas in 2013, the majority who experienced CHE had sought care from a private provider.

### Determinants of catastrophic health expenditure

First, we fitted a logistic regression model to examine the factors associated with CHE in 2007 and 2013. The results are presented in the S2 Table. The results show that, the determinants of facing CHE were similar but there were more significant factors in 2013 as compared to 2007.

In 2007, richer households (OR = 0.10; 95% CI, 0.05–0.20), employed households’ heads (OR = 0.60; 95% CI, 0.445–0.808), and female-headed households(OR = 0.65; 95% CI, 0.47–0.91) lowered the odds of incurring CHE, whereas seeking care from private providers (OR = 1.69; 95% CI, 1.26–2.27) and households with a chronically ill member (OR = 1.37; 95% CI, 1.02–1.89) increased the odds of incurring CHE.

The results further show that in 2013, richer households (OR = 0.02; 95% CI, 0.01–0.05), employed household heads (OR = 0.70; 95% CI, 0.51–0.96), medium-sized households (OR = 0.50; 95% CI, 0.35–0.73), and larger households (OR = 0.43; 95% CI, 0.24–0.75) lowered the odds of incurring CHE. Meanwhile, seeking care from private providers (OR = 2.90; 95% CI, 2.13–3.95), households with an elderly member (OR = 1.58; 95% CI, 0.19–2.30), insured households (OR = 2.55; 95% CI, 1.73–3.77), and households with a chronically ill member (OR = 1.93; 95% CI, 1.45–2.57) increased the odds of incurring CHE.

### Inequalities in catastrophic health expenditure

Table 2 presents analysis of the concentration indices for CHE in 2007 and 2013. The analysis shows an increase in the concentration index of CHE by 38.7%, from -0.271 in 2007 to -0.376 in 2013. The negative concentration indices reveal that CHE is more concentrated among the less well-off (poor), meaning that the poor are more likely to incur CHE than the rich in Kenya.

**Table 2:** Concentration indices for catastrophic health expenditure, 2007 and 2013.

Year	Concentration Index (CI)	Robust Std. error	P Value
2007	-0.271	0.036	<0.001***
2013	-0.376	0.028	<0.001***
CI _2013_—CI _2007_	-0.105		

**Fig 2:**
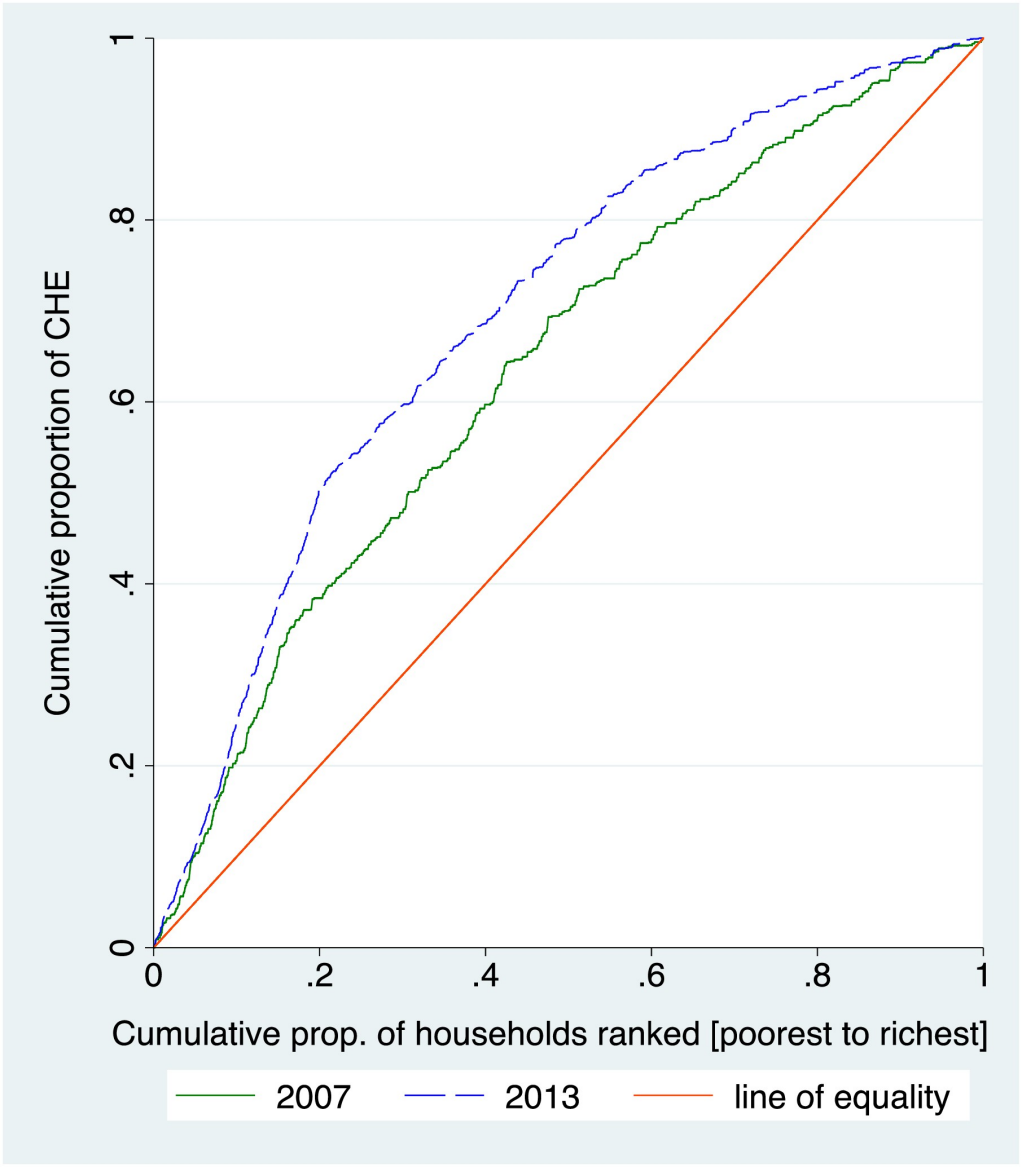
Concentration curves for catastrophic health expenditure, 2007 and 2013.

The concentration curves in Fig 2 demonstrates the same, as they lie above the line of equality. This further shows that poor households spent a higher share of their household expenditure on healthcare services in 2007 and 2013 as compared to rich households.

### Decomposition of socioeconomic inequalities in catastrophic health expenditure

We decomposed the CHE concentration index against the socioeconomic variables to show the relative contribution of each variable to inequalities in CHE, as shown in Table 3. The table presents the concentration index for each of the variables, the elasticity, the absolute contribution as well as the percentage contribution of each variable to the inequalities in CHE.

**Table 3:** Decomposition of inequalities in catastrophic health expenditure, 2007 and 2013.

Variables	2007				2013			
	Elasticity	Con. Index	Absolute %		Elasticity	Con. Index	Absolute %	
			Contr.	Contr.			Contr.	Contr.
**Wealth status (Ref. = Poorest)**								
Second quintile	-0.185	-0.398	0.074	-27.2%	-0.321	-0.399	0.128	-30.5%
Middle quintile	-0.214	0.030	-0.006	2.4%	-0.357	0.000	0.000	0.0%
Fourth quintile	-0.263	0.434	-0.114	42.1%	-0.482	0.400	-0.192	51.2%
Richest quintile	-0.284	0.817	-0.232	85.5%	-0.576	0.800	-0.461	122.4%
**Female headed household**	-0.064	-0.156	0.010	-3.7%	-0.051	-0.072	0.004	0.9%
**Age group of household head(Ref. = Below 25 years)**								
25–40 years	0.020	0.060	0.001	-0.5%	0.005	0.006	0.000	-1.3%
40 + years	0.005	-0.038	0.000	0.1%	0.042	-0.007	0.000	1.0%
**Education of household head (Ref. = No education)**								
Primary education	-0.133	-0.093	0.012	-4.6%	-0.028	-0.166	0.004	-1.1%
Secondary education	-0.089	0.243	-0.021	7.9%	-0.021	0.175	-0.003	0.0%
Tertiary education	-0.016	0.625	-0.009	3.6%	-0.007	0.548	-0.004	0.1%
**Employed Household head**	-0.335	0.046	-0.015	5.6%	-0.931	0.026	-0.024	6.4%
**Household wt. U5 Children**	-0.012	-0.002	0.000	0.0%	-0.064	-0.069	-0.004	-1.2%
**Household wt. Elderly (60+)**	0.024	-0.156	-0.004	1.4%	-0.105	-0.103	0.005	1.3%
**Household size (Ref. = 1–3 Small size)**								
4–6 Medium size	-0.093	0.035	-0.003	1.2%	-0.065	0.001	-0.001	0.3%
7+ Large size	0.016	0.039	0.001	-0.2%	-0.135	-0.084	0.011	-3.0%
**Urban residence**	-0.037	0.396	-0.015	5.5%	0.072	0.262	0.019	-5.1%
**Insured households**	0.017	0.105	0.002	-0.7%	0.214	0.392	0.084	-22.3%
**Type of health provider (Ref. = Public providers)**								
Private providers	0.141	0.106	0.015	-5.5%	0.257	0.153	0.039	-10.5%
Other providers	-0.028	0.008	0.000	0.1%	0.010	-0.039	0.000	0.1%
**Household wt. member wt. chronic illness**	0.101	0.017	0.002	-0.7%	0.194	-0.016	-0.003	-0.9%
Residual			0.031	-11.4%			0.022	-5.9%

Contr. refers to Contribution; Con. Index refers to Concentration index (CI).

The results show that, in 2007, the higher wealth quintiles, employed household heads, higher education status, and urban residence were positive contributors to inequality, meaning they favoured the well-off but disadvantaged the poor. Meanwhile, the insured households and private health providers, were the main negative contributors, implying they favoured the poor but disadvantaged the well-off.

In 2013, higher wealth quintiles and employed households’ heads were the main positive contributors to inequality, implying they favoured the well-off, whereas insured household heads, and private health providers were negative contributors, meaning they favoured the worse-off.

Further, the results show that variables such as age of the household head, households with children under five, and seeking care from other providers in 2007 and age of the household head and medium-sized households in 2013 had minimal contribution to inequality, implying it was less sensitive to these variables. The unexplained factors were negative contributors of inequality, suggesting they tend to favour the worse-off, with their contribution to inequality decreasing from 11.4% in 2007 to 5.9% in 2013.

### Oaxaca-type decomposition of change in inequalities for catastrophic health expenditure

The decomposition of the total change in inequalities between 2007 and 2013 is presented in Table 4.

**Table 4:** Oaxaca-type decomposition of change in inequalities for catastrophic health expenditure, 2007 and 2013.

Variables	Variation 1(Eq 7)		Variation 2(Eq 8)		Total	
	ΔC.η_kt_	Δη.C_kt-1_	ΔC.η_kt-1_	Δη.C_kt_	Total	%
**Wealth status**						
Second quintile	0.001	0.054	0.000	0.054	0.055	-52.4%
Middle quintile	0.011	-0.004	0.006	0.000	0.006	-5.7%
Fourth quintile	0.016	-0.095	0.009	-0.088	-0.079	75.2%
Richest quintile	0.010	-0.239	0.005	-0.234	-0.229	218.1%
**Female head household**	-0.004	-0.002	-0.005	-0.001	-0.006	5.7%
**Education of household head**						
Primary education	0.002	-0.009	0.01	-0.017	-0.007	6.7%
Secondary education	0.002	0.017	0.007	0.012	0.018	-17.1%
Tertiary education	-0.001	0.014	0.001	0.012	0.014	-13.3%
**Age group of household head**						
25–40 years	0.000	-0.002	-0.002	0.000	-0.001	1.0%
40+ years	0.002	-0.001	0.000	0.000	0.001	-1.0%
**Employed Household head**	0.022	-0.029	0.008	-0.015	-0.007	6.7%
**Household wt. U5 Children**	0.004	0.000	0.001	0.004	0.005	-4.8%
**Household wt. Elderly (60+)**	0.003	-0.004	0.002	-0.002	-0.001	1.0%
**Household size**						
4–6 Medium HH	0.001	0.001	0.002	0.000	0.002	-1.9%
7+ Large HH	0.017	-0.006	-0.002	0.013	0.011	-10.5%
**Urban residence**	-0.010	0.044	0.005	0.030	0.034	-32.4%
**Insured households**	0.063	0.020	0.005	0.078	0.083	-79.0%
**Type of health provider**						
Private providers	0.012	0.013	0.006	0.018	0.025	-23.8%
Other providers	0.000	0.000	0.001	-0.002	0.000	0.0%
**Household wt. member wt. chronic illness**	0.003	0.001	0.001	0.002	0.003	-2.9%
**Totals**	0.151	-0.228	0.059	-0.138	-0.076	72.4%
**Residual**					-0.029	27.6%
Difference (CI_t_−CI_t-1_)					-0.105	100.0%

E: Elasticity, CI: Concentration Index, t:2013, (t-1):2007; Eq: Equation.

Overall, there was -0.105(39%) increase in socioeconomic equality in CHE between 2007 and 2013. The observed socioeconomic characteristics accounted for 72.4% of the change in inequalities in CHE, while the remaining 27.6% was due to unobserved characteristics (residual).

High wealth quintile was the main contributor to the change in inequalities between 2007 and 2013, contributing to 218.1% increase. We observe that it is the change in elasticities of the wealth status that accounted for the significant increase in CHE inequalities, the bulk being attributable to the high wealth quintile. Insurance status of the household was the second variable that accounted for the most significant change in CHE inequalities, contributing 79% to lessening the inequalities. Other variables that contributed to lessening the inequalities included urban residence (32.4%), private health providers (23.8%), education level of the household head (17.1% and 13.3%), and larger household size (10.5%).

## Discussion

The results show a decline in the incidence of CHE between 2007 and 2013, which is consistent with the national report in Kenya [41]. Determinants that lowered the odds of incurring CHE included higher wealth status, employed household heads and larger households. In the Sub-Saharan region, people with upper economic status, households with employed heads, and larger households are less likely to experience financial burden [9,42]. Seeking care from a private provider and having a household member with chronic illness increased the odds of incurring CHE. It can be inferred that this is because private health care providers charge high fees [43] and chronic illnesses are associated with long-term treatment costs [44]. Contrary to expectation, households with insurance increased the odds of incurring CHE in 2013. This is likely because the NHIF did not cover outpatient services by 2013 when both studies were conducted [23]. Additionally, in 2013, the insured had a higher utilisation rate for inpatient services, compared to the uninsured [41]. Furthermore, evidence suggests that health insurance in itself can increase the risk of high catastrophic spending by encouraging individuals to seek care from high-level providers [45,46].

Despite the decrease in the incidence of CHE, inequalities in CHE increased overtime. This implies that socioeconomic inequalities in catastrophic expenditure have worsened in Kenya. This is corroborated by other studies in Kenya that shows inequalities in CHE still exist and are disproportionately concentrated amongst the worse-off [13,23]. Furthermore, the results show that the better-off experienced a higher percentage of decline in CHE compared to the worse-off.

Higher wealth quintiles and employed household head were the main contributors of inequalities in CHE in both 2007 and 2013. Poorer households in Kenya are reported to spend more OOP in proportion to consumption than their rich counterparts [22]. The unemployed have no or little income, which restricts their access to health care [47], Insured households and private health providers contributed to pro-rich inequality in CHE; furthermore, high socioeconomic status is associated with higher odds of insurance coverage [17]. Evidence suggests overuse of health services amongst households that are insured and low utilisation amongst the non-insured [48,49]. A recent study in Kenya revealed that pro-rich inequality in health service use is significantly higher for care provided in privately owned facilities [21].

Changes in inequalities and elasticities of the socioeconomic determinants largely explain the change in socioeconomic inequalities in CHE. The analysis suggests that there are unobserved factors that contributed to the change in inequalities. Household wealth status was the main contributor to the rise in inequalities in CHE. This points to the existence of significant inequalities in Kenya not only in health but across various sectors and geographical regions. For instance, more than half (59.4%) of the country’s total expenditure is controlled by the richest quintile while the poorest quintile controls only 3.6 per cent. Also, the largest share of household expenditures is controlled by the fourth and richest quintile [50]. Changes in inequality in a household’s insurance status accounted for a substantial decrease in the change in CHE inequalities. This could be because of the decline in health insurance coverage inequalities in Kenya [17]. This underscores the critical role of health insurance coverage in reducing inequalities in the financial burden imposed by healthcare. Emphasising the marked rural-urban inequalities that exist in Kenya [51], inequalities relating to rural-urban residence was important in explaining the change in CHE inequalities. The contribution of larger household size to reducing inequalities stressed the role of social capital through the pooling of resources among several households or community members, which is vital in the African communal setting and the large informal workforce [52,53].

Education is critical in enhancing health outcomes by reducing the need for health care and associated costs of dependence [54]. Our results show that the education level of the household head was attributed to reducing inequalities overtime. This is undoubtedly due to the multiplier effect of education given educated household heads are likely to be employed and thus have better economic status and better access to health services [55]. Furthermore, our descriptive analysis indicates an improvement in the education level of the household heads between the surveys, with up to 74% in 2013, having at least primary level education.

Overall, the results underscore the existence of socioeconomic inequalities in Kenya that disadvantage the poor. In accordance with other studies, the results emphasise that the drivers of socioeconomic inequalities in healthcare payments extend to other social sectors beyond the health sector [21]. Despite Kenya showing progress in addressing poverty, the burden on the poor is still significantly exacerbated by these relatively high and persistent inequalities [50]. Collaboration across sectors such as social protection could offer more significant impact in financial risk protection for the most vulnerable hence reduce socioeconomic-related inequalities which if left unchecked could reverse the gains in the health sector. Analysis of inequalities in healthcare access is therefore vital as it unravels existing nuances and variations across socioeconomic groups. Furthermore, it is possible to have national averages decrease but mask disparities amongst subgroups [28]. For instance, as shown in this study, although the incidence of CHE declined over time, the inequalities affecting the poor have deteriorated.

There are limitations that need to be considered while interpreting the findings. First, in both surveys, data on outpatient health expenditure was collected based on the last 4 weeks preceding the survey, whereas the inpatient expenditure was annual. To have a standard period of reference, we annualised the outpatient expenditure as per practice. This could possibly overestimate or underestimate healthcare expenditures. Second, the expenditure in the surveys are all self-reported and may suffer from recall bias. Third, the timing of the study and seasonality may have implications on the type of illness and health-seeking behaviour, given that some illnesses are prevalent at certain times of the year, subsequently having implications on the cost burden at the household level [56].

## Conclusions

Socioeconomic inequalities in CHE persist in Kenya, suggesting that the existing financial risk protection mechanisms have not sufficiently addressed these disparities. The recent changes to the National Social Health Insurance Fund (NSHIF) is a positive move, but it is based on a premium; thus, many poor households may still lack the ability to pay. There are still opportunities to improve on pro-poor mechanisms through the UHC initiatives hence address socioeconomic inequalities in the utilisation of healthcare services in Kenya.

This study demonstrates that understanding the socioeconomic factors that sustain inequalities in CHE is paramount in informing policymakers of the need to intensify and tailor pro-poor interventions. A fundamental lesson from the findings is that the drivers of inequalities, such as economic status and unemployment, extend beyond the health sector. Therefore, a multi-sectoral approach should be considered in addressing socioeconomic inequalities so as to draw synergies and efficiencies across various sectors hence accelerate the achievement of UHC.

## Supporting information

S1 Table This table summarises the analyses of the incidence of catastrophic health expenditure by wealth quintiles in 2007 and 2013. (DOCX)

S2 Table This table summarises the results of the regression model for the determinants of catastrophic health expenditure for 2007 and 2013. (DOCX)
