# Supplementary material for: Decomposition of changes in socioeconomic inequalities in catastrophic health expenditure in Kenya
Source: PLoS One. 2020 Dec 29;15(12):e0244428. doi: 10.1371/journal.pone.0244428 (PMC7771691; doi:10.1371/journal.pone.0244428)
Supplement: S1 Table — (DOCX) [file pone.0244428.s001.docx]

**S1 Table**: Households that experienced Catastrophic Health Expenditure (CHE) by Wealth Quintiles, 2007 and 2013

| **Description** | **Year** | ***Households with CHE*** | | ***Incidence difference (2013-2007)*** | | ***Households without CHE*** | | **Total Cases(N)** |
| --- | --- | --- | --- | --- | --- | --- | --- | --- |
|  |  | **Cases**  **(N)** | **Percent* (%)** | **Absolute diff.** | **Percent diff.(%)** | **Cases**  **(N)** | **Percent (%)** |  |
| Total  (All quintiles) | 2007 | 427 | 11.4 | -4.89 | 42.7 | 3,301 | 88.6 | 3,728 |
|  | 2013 | 1,084 | 6.5 |  |  | 15,442 | 93.4 | 16,526 |
| Richest Quintile | 2007 | 37 | 4.9 | -3.09 | 63.3 | 721 | 95.1 | 758 |
|  | 2013 | 68 | 1.8 |  |  | 3,723 | 98.2 | 3,791 |
| Fourth Quintile | 2007 | 55 | 7.2 | -4.55 | 63.5 | 712 | 92.8 | 767 |
|  | 2013 | 92 | 2.6 |  |  | 3,420 | 97.4 | 3,512 |
| Middle Quintile | 2007 | 74 | 9.8 | -4.99 | 50.9 | 680 | 90.2 | 754 |
|  | 2013 | 152 | 4.8 |  |  | 3,001 | 95.2 | 3,153 |
| Second Quintile | 2007 | 99 | 12.8 | -5.64 | 44.0 | 673 | 87.2 | 772 |
|  | 2013 | 225 | 7.2 |  |  | 2,909 | 92.8 | 3,134 |
| Poorest Quintile | 2007 | 162 | 23.9 | -5.30 | 22.1 | 515 | 76.1 | 677 |
|  | 2013 | 547 | 18.6 |  |  | 2,389 | 81.4 | 2,936 |

**Incidence of catastrophic health expenditure (CHE) at 40% threshold; diff: Difference*
