# Supplementary material for: Decomposition of changes in socioeconomic inequalities in catastrophic health expenditure in Kenya
Source: PLoS One. 2020 Dec 29;15(12):e0244428. doi: 10.1371/journal.pone.0244428 (PMC7771691; doi:10.1371/journal.pone.0244428)
Supplement: S2 Table — (DOCX) [file pone.0244428.s002.docx]

**S2 Table:** Multivariate regression model for determinants of catastrophic health expenditure, 2007 and 2013

| **Variables** | **2007** | | | **2013** | |  |
| --- | --- | --- | --- | --- | --- | --- |
|  | **Odds Ratio** | **[95% Conf. Interval]** | | **Odds Ratio** | **[95% Conf. Interval]** |  |
| **Wealth status (Ref. Poorest)** |  |  | |  |  |  |
| Second quintile | 0.454*** | (0.321- 0.644) | | 0.282*** | (0.228-0.484) |  |
| Middle quintile | 0.284*** | (0.190 - 0.425) | | 0.159*** | (0.106-0.255) |  |
| Fourth quintile | 0.182*** | (0.112 - 0.295) | | 0.063*** | (0.057-0.143) |  |
| Richest quintile | 0.105*** | (0.055 - 0.200) | | 0.025*** | (0.015-0.050) |  |
| **Gender of household head**  **(Ref. Male)** | | | |  |  |  |
| Female | 0.656** | (0.473-0.908) | | 1.005 | (0.748-1.350) |  |
| **Age of household head**  **(Ref. Below 25 years)** | | | |  |  |  |
| 25-40 years | 1.311 | (0.635 - 2 .707) | | 1.258 | (0.612-2.585) |  |
| 40+ years | 1.205 | (0.574 - 2.526) | | 0.860 | (0.406-1.818) |  |
| **Education level of the household head**  **(Ref. No Education)** | | | |  |  |  |
| Primary Education | 0.764 | (0.546-1.069) | | 1.449 | (0.836-2.510) |  |
| Secondary Education | 0.744 | (0.468-1.184) | | 1.150 | (0.588-2.246) |  |
| Tertiary Education | 0.772 | (0.342-1.742) | | 2.203 | (0.955-5.083) |  |
| **Employment status of the HH head**  **(Ref. Unemployed HH head)** | | | |  | **.** |  |
| Employed HH head | 0.600*** | (0.445-0.808) | | 0.703** | (0.512-0.963) |  |
| **Household wt. U5 Children**  **(Ref. Above 5 years)** | | | |  |  |  |
| Less than 5 (< 5) years | 0.896 | (0.642-1.251) | | 0.882 | (0.591-1.317) |  |
| **Household wt. Elderly**  **(Ref. < 60 years)** | | | |  |  |  |
| 60+ years | 1.174 | (0.839-1.643) | | 1.586** | (1.090-2.308) |  |
| **Household size**  **(Ref. 1-3 Small HH)** | | | |  |  |  |
| 4-6 Medium HH | 0.742 | (0.528-1.043) | | 0.508*** | (0.354-0.730) |  |
| 7+ Large HH | 1.001 | (0.648-1.546) | | 0.432*** | (0.248-0.754) |  |
| **Residence (Ref. Rural residence)** |  |  |  |  |  |  |
| Urban Residence | 0.815 | (0.543-1.223) | | 0.976 | (0.715-1.333) |  |
| **Insurance status**  **(Ref. Not insured)** | | | |  |  |  |
| Insured Households | 1.263 | (0.883-1.805) | | 2.558*** | (1.734-3.773) |  |
| **Type of health provider**  **(Ref. Public)** | | | |  |  |  |
| Private provider | 1.694*** | (1.260-2.277) | | 2.903*** | (2.131-3.954) |  |
| Other providers | 0.705 | (0.458 -1.087) | | 0.737 | (0.478-1.136) |  |
| **HH wt. member with chronic illness**  **(Ref. No chronic illness)** | | | |  |  |  |
| Member with Chronic illness | 1.377** | (1.025-1.849) | | 1.934*** | (1.451-2.579) |  |
| _cons | 0.421 | (0.188-0.947) | | 0.154 | (0.061-0.391) |  |
| **** p<0.01, ** p<0.05* | | | | | | |
